# Exploring host–pathogen interactions in the *Dictyostelium discoideum–Mycobacterium marinum* infection model of tuberculosis

**DOI:** 10.1242/dmm.050698

**Published:** 2024-07-22

**Authors:** Sandra Guallar-Garrido, Thierry Soldati

**Affiliations:** Department of Biochemistry, Faculty of Science, University of Geneva, 30 quai Ernest-Ansermet, Science II, 1211 Geneva-4, Switzerland

**Keywords:** Autophagy, *Dictyostelium*, ESCRT, Mycobacteria, Phagocytes

## Abstract

*Mycobacterium tuberculosis* is a pathogenic mycobacterium that causes tuberculosis. Tuberculosis is a significant global health concern that poses numerous clinical challenges, particularly in terms of finding effective treatments for patients. Throughout evolution, host immune cells have developed cell-autonomous defence strategies to restrain and eliminate mycobacteria. Concurrently, mycobacteria have evolved an array of virulence factors to counteract these host defences, resulting in a dynamic interaction between host and pathogen. Here, we review recent findings, including those arising from the use of the amoeba *Dictyostelium discoideum* as a model to investigate key mycobacterial infection pathways. *D. discoideum* serves as a scalable and genetically tractable model for human phagocytes, providing valuable insights into the intricate mechanisms of host–pathogen interactions. We also highlight certain similarities between *M. tuberculosis* and *Mycobacterium marinum*, and the use of *M. marinum* to more safely investigate mycobacteria in *D. discoideum*.

## Teaser

**Summary:** Pathogenic mycobacteria establish infections by evading host immune defences. The amoeba *Dictyostelium discoideum* infected with *Mycobacterium marinum* is an innovative model to investigate key mycobacterial virulence pathways and host responses.

## Introduction

*Mycobacterium tuberculosis* (Mtb) causes tuberculosis (TB), a disease responsible for approximately 10 million cases and approximately 1.5 million annual deaths worldwide (WHO, 2023). Indeed, in 2022, TB ranked as the second most deadly infectious disease globally, following COVID-19 and surpassing HIV/AIDS (WHO, 2023). Mtb belongs to the genus *Mycobacterium*, which also includes a substantial group of nontuberculous mycobacteria of medical significance, such as *Mycobacterium marinum* (Mm), *Mycobacterium leprae*, *Mycobacterium abscessus* or *Mycobacterium avium* complex (reviewed by Sharma and Upadhyay, 2020).

**Fig. 1.**
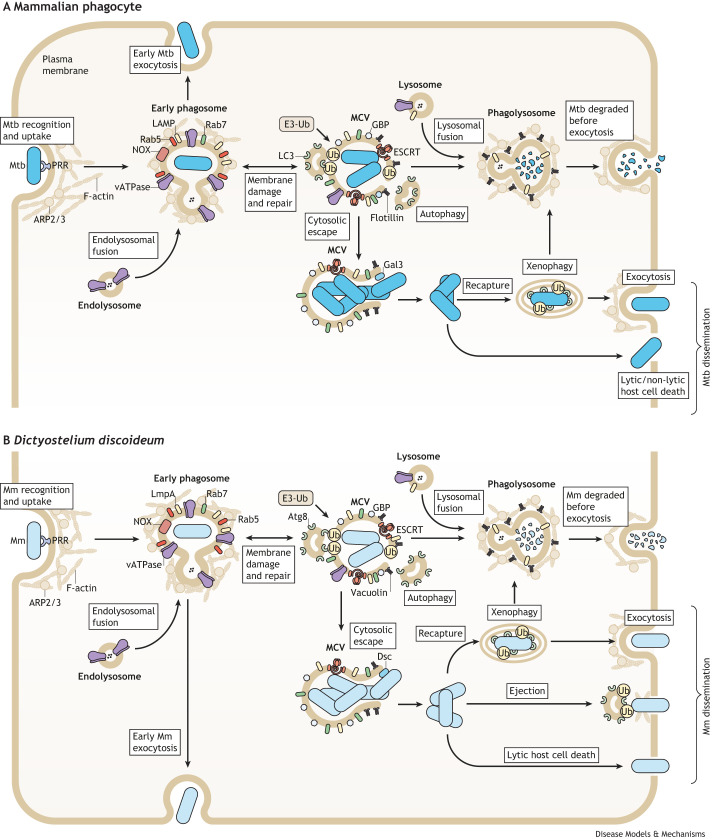
**Infection cycle of a pathogenic mycobacterium in mammalian phagocytes and in the amoeba *Dictyostelium discoideum*.** Schematics of the phagocytosis of pathogenic *Mycobacterium tuberculosis* (Mtb) in a mammalian phagocyte (A) and *Mycobacterium marinum* (Mm) in the amoeba *Dictyostelium discoideum* (Dd) (B). Mycobacteria are recognized by host pathogen recognition receptors (PRRs) on the cell surface, then the host cells internalize the pathogens via phagocytic cups, which are supported by an F-actin scaffold and actin-related protein (ARP) 2/3 complexes. Early phagosomes undergo maturation, transitioning from Ras-related proteins 5 (Rab5) to 7 (Rab7), acquiring vacuolar ATPase (vATPase) to decrease luminal pH, lysosomal enzymes and lysosome-associated membrane proteins (LAMP/LmpA) through endosome fusion, and activating the NADPH oxidase (NOX) complex. In mammalian macrophages and Dd, ingested mycobacteria impede phagosome maturation by forming a mycobacteria-containing vacuole (MCV), within which they proliferate (Barisch et al., 2015; Cardenal-Muñoz et al., 2017) and induce membrane damage via virulence factors that can take advantage of host membrane microdomains, such as those containing flotillins/vacuolins. Alternatively, mycobacteria can undergo early exocytosis before phagosome maturation or replication within the MCV. The host endosomal sorting complex required for transport (ESCRT) machinery and autophagy can repair the damaged MCV membrane, and host factors, such as guanylate-binding protein (GBP), bind the MCV to limit mycobacterial growth. This ultimately leads to mycobacterial degradation upon lysosome fusion with the MCV. However, a dynamic state can be created that alternates between mycobacterial damage and host repair (Lopez-Jimenez et al., 2018; Mehra et al., 2013). Extensive damage to the MCV membrane can recruit the damage sensors galectin-3 (Gal3) in mammalian cells and discoidins (Dsc) in Dd, and can lead to cytosolic access for mycobacteria, facilitating their ubiquitination by E3 ubiquitin ligases (E3-Ub), recapture and subsequent degradation through xenophagy (Cardenal-Muñoz et al., 2017; Gutierrez and Enninga, 2022). Mycobacteria may win over host cell defences, resulting in cytosolic replication and dissemination via exocytosis, host cell lytic/non-lytic mechanisms, or by ejection in the case of Dd (Bo et al., 2023; Gerstenmaier et al., 2024, 2015; Hagedorn et al., 2009). These processes can lead to the formation of extracellular aggregates, which are then phagocytosed by newly recruited Dd (Hagedorn et al., 2009) and macrophages, significantly contributing to bacterial replication, growth, and dissemination (Toniolo et al., 2023). Abbreviations: Atg8, autophagy-related protein 8; LC3, microtubule-associated proteins 1A/1B light chain 3B (MAP1LC3B); Ub, ubiquitinated proteins.

TB presents a complex and persistent clinical challenge across multiple stages of the disease. Lung macrophages are initially infected by Mtb, which then serve as the primary replication site, significantly contributing to bacterial dissemination. Macrophages typically engulf, kill and digest pathogens via phagocytosis (see Glossary, Box 1) and xenophagy (Box 1) (Fig. 1). However, certain bacteria, including Mtb and Mm, adeptly manipulate phagocytic cells, creating permissive environments for their growth and dissemination (Cardenal-Munoz et al., 2018). To better understand and to facilitate TB research, researchers study mycobacteria that are less harmful to humans, including Mm, which shares virulence factors with Mtb (Tobin and Ramakrishnan, 2008).

The complex interplay between mycobacteria and cell-autonomous defence mechanisms has been extensively studied in a variety of model systems, including in amoebae (Cardenal-Munoz et al., 2018), zebrafish (Ramakrishnan, 2013), *Drosophila* (Marshall and Dionne, 2022), mice (Li and Li, 2023), human primary bone marrow-derived macrophages (BMDMs) (Podinovskaia et al., 2013) and human induced pluripotent stem cell-derived macrophages (iPSDMs) (Bernard et al., 2021). Cell models, in particular, are advancing our understanding of the molecular, cellular and dynamic aspects of the interactions that occur between host cells and mycobacteria, such as Mm and Mtb, which we discuss here.

We also review here the use of the amoeba *Dictyostelium discoideum* (Dd) as a model to explore cell-autonomous defence mechanisms that occur in response to mycobacterial infections. Dd shares high levels of evolutionary conservation of host defence mechanisms with mammalian cells and, as such, has proven to be a powerful model for studying host–pathogen interactions and for identifying metabolic pathways relevant to macrophages, as detailed throughout the Review (Cardenal-Munoz et al., 2018; Dunn et al., 2018).

**Box 1. Glossary**

**Autophagy:** a conserved intracellular degradation pathway that captures damaged cytoplasmic materials or cytosolic pathogens within an autophagosome – a double-membrane organelle, which fuses with lysosomes, forming a degradative environment known as the autolysosome, in which targeted material undergoes digestion.

**DNA extracellular traps:** structures released by immune cells, consisting of chromatin and antimicrobial proteins, to ensnare and neutralize pathogens during infections.

**Endosomal sorting complex required for transport (ESCRT):** an evolutionarily conserved multi-protein complex involved in membrane dynamics and repair that consists of ESCRT-0, ESCRT-I, ESCRT-II, ESCRT-III, Vps4 (AAA-ATPase) and accessory proteins such as ALIX.

**Flotillins:** membrane proteins associated with lipid rafts in cell membranes, playing roles in cellular processes such as signal transduction, endocytosis and membrane trafficking.

**Galectins:** soluble β-galactoside-binding receptors that sense self and non-self carbohydrates. Fifteen mammalian galectins act extracellularly and intracellularly.

**Guanylate-binding proteins (GBPs):** proteins belonging to an interferon γ-inducible subfamily of guanosine triphosphatases (GTPases) that are produced in several cell types, including macrophages, and play a role in the immune response.

**Inflammasome system:** a multiprotein complex that detects pathogen-associated or danger signals, triggering inflammation and the release of pro-inflammatory cytokines, crucial for immune responses.

**iNOS:** inducible nitric oxide synthase, an enzyme involved in the production of the antimycobacterial molecule nitric oxide (NO) from L-arginine.

**Kil1:** a Golgi sulfotransferase involved in the maturation of lysosomal enzymes.

**Kil2:** a putative magnesium pump involved in the inflammasome system.

**Lysozymes:** antimicrobial enzymes, including glycosidases, proteases, nucleases, lipases, phosphatases and sulfatases.

**Mycobacteria-containing vacuole (MCV):** a specialized compartment within host cells where mycobacteria reside after manipulating phagosomes, allowing them to evade host defences and manipulate cellular processes for survival and replication.

**Necroptosis:** a programmed form of cell death characterized by plasma membrane rupture and release of cellular contents, triggered by specific signalling pathways.

**Pathogen-associated molecular patterns (PAMPs):** conserved molecules found in pathogens, recognized by pattern recognition receptors, initiating immune responses.

**Phagosome:** cellular compartment formed by the engulfment of particles or microorganisms, serving to degrade and digest the ingested material via fusion with lysosomes.

**Phagocytosis:** cellular process in which specialized cells engulf and internalize pathogens or cellular debris into membrane-bound vesicles called phagosomes, facilitating their degradation and clearance.

**Pyroptosis:** a highly inflammatory form of programmed cell death initiated by inflammasome activation, leading to membrane rupture and release of pro-inflammatory cytokines.

**Siderophores:** molecules secreted by microorganisms to scavenge iron from the environment.

**Stress granules:** aggregations of non-translated messenger ribonucleoproteins and diverse proteins, often regarded as membraneless organelles that can associate with membranes.

**Target of rapamycin complex 1 (TORC1):** a nutrient-sensitive kinase complex that modulates cellular responses based on nutrient availability.

**Ubiquitination:** a post-translational modification in which ubiquitin binds covalently to lysine residues in target proteins. Ubiquitination can trigger various cell responses, including protein degradation via the proteasome, autophagy and innate immune signalling.

**Xenophagy:** selective autophagy that targets cytosolic bacteria by forming autophagosomes, encapsulating the bacteria, and facilitating their eventual delivery to lysosomes.

## Mtb and Mm share conserved virulence strategies

Using Mm as an Mtb research model entails benefits, such as genetic resemblance and reduced risk to laboratory staff, but also several constraints, which are discussed below. Mm is a close genetic relative of Mtb, sharing significant genomic similarity (Stinear et al., 2008) and essential genes (Lefrançois et al., 2024). At the proteome level, Mtb and Mm share ∼3000 orthologous proteins, displaying an average amino acid identity of 85% (Stinear et al., 2008).

Mm causes skin granulomatous infections in humans and TB-like infections in poikilotherms, such as frogs and fish, whereas Mtb infects humans, causing TB (Davis et al., 2002). Consequently, Mm presents itself as an ideal organism that could be used to explore the mechanisms of Mtb virulence in a safer and more efficient manner given the stringent safety measures that are required when conducting research using Mtb. However, evolutionary divergence between both mycobacteria species may lead to different host–pathogen interactions, immune evasion mechanisms or even antimicrobial drug responses, implying that findings in Mm might not always directly translate to Mtb.

The infection process orchestrated by Mtb and Mm in both macrophages and Dd is highly similar (Fig. 1). Both Mtb and Mm employ similar strategies to evade host cell defence mechanisms. These include the release of proteinaceous and lipidic virulence factors and the creation of a mycobacteria-containing vacuole (MCV, Box 1) to bypass phagolysosome maturation and to escape to the host cytosol. Notably, both species harbour the type VII secretion system ESX-1, which is encoded by the ‘region of difference 1’ (RD1) locus and is responsible for secreting the EsxA–EsxB dimer that is crucial for damaging the MCV (Osman et al., 2022; Smith et al., 2008). MCV damage favours escape of Mm and Mtb to the cytosol and release of Mtb DNA into the cytosol, prompting the host to produce type I interferons (Watson et al., 2015). It is worth noting that the attenuated *Mycobacterium bovis* bacillus Calmette–Guérin (BCG) strain lacks RD1 and is used as a vaccine against Mtb (Conrad et al., 2017; Schnettger et al., 2017). Moreover, Mtb and Mm in which RD1 is deleted (ΔRD1) exhibit restricted growth in amoebae (Lopez-Jimenez et al., 2018), mouse or human macrophages (Lewis et al., 2003), iPSDMs (Bernard et al., 2021) and human monocyte-derived macrophages (hMDMs) (Welin et al., 2011). Additionally, both Mm and Mtb possess the ESX-3 system, which secretes small virulence factors such as the dimer EsxG–EsxH, which can interact with the host ‘endosomal sorting complex required for transport’ (ESCRT) complex (Box 1) (Saelens et al., 2022), and the bacterial ESX-5 system (Abdallah et al., 2008). Moreover, Mtb and Mm contain non-essential secretion systems, such as SecA2 (Ge et al., 2022; Serene et al., 2024; van der Woude et al., 2014; Zulauf et al., 2018). It is important to note that although the infectious processes of both mycobacteria are highly similar, there are notable differences. Mm infection thrives at 25°C, contrasting with Mtb preference for 37°C, aligning with their niche-specific adaptation.

**Fig. 2.**
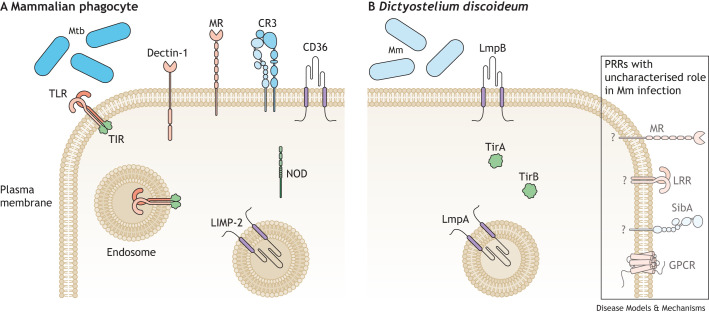
**Key receptors that recognize and uptake mycobacteria in mammalian phagocytes and *Dictyostelium discoideum*.** (A) Phagocytes, including macrophages, dendritic cells and neutrophils, of the innate immune system can detect pathogens via pathogen recognition receptors (PRRs). Among the most relevant PRRs in mammalian cells during *Mycobacterium tuberculosis* (Mtb) infection are: Toll-like receptors (TLRs) formed by leucine-rich repeats (LRRs) and Toll/interleukin-1 receptor (TIR) domains; C-type lectins [dectin-1 and mannose receptor (MR)]; class B scavenger receptor family (CD36 and LIMP-2); the integrin CR3, which can be found at the plasma membrane and in endosomal membranes; and the cytosolic nucleotide-binding oligomerization domain receptors (NODs) (Aqdas et al., 2021; Goyal et al., 2016; Gunther and Seyfert, 2018). There are several types of TLRs in mammalian phagocytes. (B) In terms of direct orthologs of these PRRs in *Dictyostelium discoideum* (Dd), only class B scavenger receptors and cytosolic proteins containing the TIR domain have been identified and studied in the context of *Mycobacterium marinum* (Mm) infection (Chen et al., 2007; Sattler et al., 2018). Dd harbours lysosomal membrane proteins A (LmpA) and B (LmpB) that serve as scavenger receptors, akin to LIMP-2 and CD36 in mammals, respectively. Moreover, Dd expresses two cytosolic proteins containing the TIR domain, TirA and TirB (Li et al., 2009). TirA is crucial for effective phagocytosis of Gram-negative bacteria (Chen et al., 2007; Zhang et al., 2016b). Dd also has 45 LRR transmembrane proteins without a cytosolic TIR domain, which have been described but not further studied. Dd also possesses three orthologs of C-type lectin receptors (MR), but their functions necessitate further examination (Cosson and Soldati, 2008). Dd also expresses ‘similar to integrin-β A’ (SibA), which shares characteristics with mammalian CR3 and is involved in adhesion and phagocytosis. Dd features additional receptors among which Far1, a Venus-trap G protein-coupled receptor (GPCR), is implicated in binding bacterial pathogen-associated molecular patterns (PAMPs) (Xu et al., 2021).

Mycobacterial species are characterized by their complex cell walls, which consist of a core layer made up of peptidoglycan, arabinogalactan and specific mycolic acids, the composition of which varies depending on the species (Chiaradia et al., 2017). Peptidoglycan and mycolic acids are significant pathogen-associated molecular patterns (PAMPs, Box 1) (Hossain and Norazmi, 2013); yet, limited research has been conducted on arabinogalactan due to the unavailability of arabinogalactan-deficient mycobacteria (Toyonaga et al., 2016). Recent studies have used chemically synthesized arabinogalactan to demonstrate its role as a virulence factor that interacts with host galectin-9 (LGALS9) (Box 1), exacerbating mycobacterial infections in both Mtb-infected severe combined immunodeficient (SCID) mice and in Mm-infected zebrafish (Wu et al., 2021). Mycobacteria species also produce various other lipids (Guallar-Garrido et al., 2022, 2021). Host cells recognize each lipid through specific receptors (Fig. 2), as reviewed recently by Zihad et al. (2023). For instance, trehalose 6,6′-dimycolate (TDM), present in all known mycobacteria species, can be recognized by host C-type lectin or scavenger receptors, triggering an inflammatory response (Ishikawa et al., 2009) and inhibiting phagosome (Box 1) maturation (Patin et al., 2017; Spargo et al., 1991). Lipoarabinomannan (LAM) produced by species such as Mtb or Mm is recognized by host mannose and DC-SIGN (also known as CD209) receptors, exerting an anti-inflammatory effect (Maeda et al., 2003). Additionally, LAM produced by Mtb can insert into host cell membrane rafts, modifying kinase activity and impeding phagosome maturation (Welin et al., 2008). Sulfoglycolipid-1 (SL-1), which is produced only by pathogenic mycobacteria, remodels host cell membranes, impacting their fluidity and modifying autophagy (Box 1) (Gilmore et al., 2012; Bah et al., 2020). Furthermore, phthiocerol dimycocerosates (PDIMs) insert into host membranes, modifying cholesterol-enriched domains and affecting pathogenesis in zebrafish (Cambier et al., 2020), host interaction in hMDMs (Augenstreich et al., 2017), autophagy in human macrophages (Bah et al., 2020) and antibiotic resistance in nutrient-limited conditions *in vitro* (Block et al., 2023).

Additionally, mycobacteria can synthesize intracytosolic lipid inclusions that serve as energy reserves (Foulon et al., 2022), even in the absence of external stressors (Campo-Perez et al., 2022; Fines et al., 2023 preprint; Barisch and Soldati, 2017). Table 1 summarizes the virulence factors of Mtb and Mm, showcasing their fundamental structural and component similarities for efficient pathogenesis and host interactions, despite inhabiting distinct ecological niches (Tobin and Ramakrishnan, 2008).

**Table 1.**
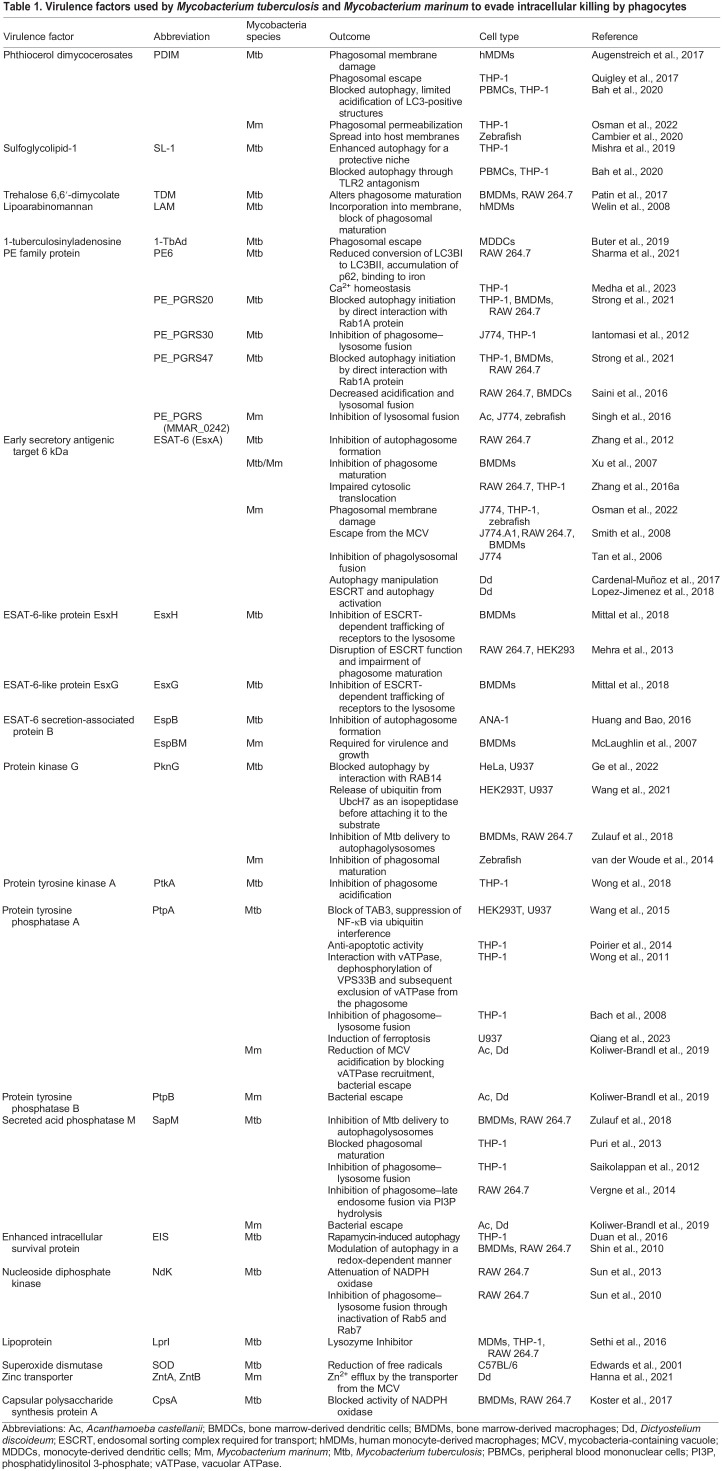
Virulence factors used by *Mycobacterium tuberculosis* and *Mycobacterium marinum* to evade intracellular killing by phagocytes

Overall, the utilization of various infection models has been crucial in elucidating the behaviour and virulence factors of mycobacteria. Specifically, Dd provides experimental advantages to study host–pathogen interactions owing to its phagocytic capabilities and conserved innate immune pathways, as elaborated in the subsequent section.

## Dd – a versatile phagocyte

Dd, a member of the Amoebozoa phylum, diverged from fungi and animals shortly after the separation of the phylum from plants (Eichinger et al., 2005). Over the past half-century, this soil amoeba has become a versatile model for studying the molecular and cellular mechanisms of cell-autonomous defence mechanisms. It has a haploid genome facilitating its genetic tractability, and low mutation rates compared to other eukaryotes (Gill and Chain, 2023). Dd is especially suited for investigating chemotaxis, cell motility, cell–cell interactions, phagocytosis, cell-autonomous immune defences and lipid-related host–pathogen interactions (Du et al., 2013), and for screening anti-infective compounds (Hanna et al., 2021).

Importantly, Dd utilizes diverse antibacterial mechanisms similar to those used by human phagocytes (Crespo-Yanez et al., 2023), making it a valuable tool for studying a variety of human pathogens, including mycobacteria (Butler et al., 2020; Lefrancois et al., 2024), *Legionella pneumophila*, *Vibrio cholera*, *Francisella noatunensis*, *Pseudomonas aeruginosa* and *Salmonella enterica*, as well as yeasts and fungi like *Cryptococcus neoformans* and *Aspergillus fumigatus*, as reviewed by Cardenal-Munoz et al. (2018) and Dunn et al. (2018). It has also played a crucial role in screening anti-bacterial compounds against multiple microorganisms, including *Klebsiella pneumoniae* (Ifrid et al., 2022) and Mm (Hanna et al., 2021). Its use as a model host has provided insights into the impact of virulence factors produced by intracellular pathogenic mycobacteria, contributing to our understanding of host–pathogen interactions (Cardenal-Muñoz et al., 2017; Hüsler et al., 2023; Swart et al., 2018).

Beyond their immune functions, Dd cells display altruistic social behaviours. Upon starvation, the amoebae transition from a single-cell state to a multicellular slug that migrates photostatically and thermostatically to the soil surface to form a fruiting body (Kin et al., 2022). The stalk is formed of altruistically dying cells supporting a mass of spores that will later be released and germinate to initiate a new cycle (Bretschneider et al., 2016; Kin et al., 2022). Sentinel cells, which make up <1% of the multicellular slug, play a key altruistic role in protecting the slug from infection by releasing mitochondrially derived DNA extracellular traps (Box 1) to combat bacterial infections (Brock et al., 2016; Chen et al., 2007; Zhang et al., 2016b). Moreover, Dd can exclude pathogen-infected cells from early stages of multicellular development (mounds) but tolerates others, suggesting a potential microbiota-like role for some bacteria (Brock et al., 2018; Farinholt et al., 2019; Haselkorn et al., 2019; López-Jiménez et al., 2019 preprint; Nicolussi et al., 2018).

Protocols have been developed to analyse host–pathogen interactions with Dd (Arafah et al., 2013; Barisch et al., 2015), including analysis of the infection course at the single-cell level (Mottet et al., 2021), isolation and proteomic characterization of MCVs (Guého et al., 2023 preprint) and other bacteria-containing vacuoles (Manske et al., 2018; Schmolders et al., 2017), and assays for gene expression in both the host and pathogen at various infection stages (Kjellin et al., 2019; Lefrançois et al., 2023).

Although Dd shares similarities with animal macrophages, there are notable differences. As a single-celled organism, Dd amoebae possess only innate immune defences, with phagocytic receptors for various ligands and other cell-autonomous pathways (Dunn et al., 2018), as depicted in Fig. 2. Notably, Dd lacks a complex inflammasome system (Box 1) responsible for pro-inflammatory cytokine secretion (Cosson and Soldati, 2008), suggesting that Dd populations will behave differently towards (myco)bacterial infections compared to mammalian host cells. Moreover, as a multicellular organism, Mm-infected Dd slugs do not develop granulomas or TB-like disease (López-Jiménez et al., 2019 preprint), as observed in other model organisms such as zebrafish or mice, precluding the study of granuloma formation and mycobacteria dissemination. Consequently, the evolutionary distance between amoebae and mammalian cells means that findings from Dd studies may not always directly translate to human diseases.

With its strengths and limitations, Dd remains valuable and is arguably the simplest model for studying mycobacteria pathogenesis and host interactions, particularly at the cellular and molecular levels, as detailed in the following sections.

## First host–pathogen contact

The initial interaction between mycobacteria and host, together with subsequent events within the host cell, greatly shapes the infection progression. In this section, we compare and contrast key insights into the initial events that take place between mycobacteria and Dd or host macrophages.

### Receptors involved in mycobacteria recognition and uptake

Among the most relevant pathogen recognition receptors in mammalian cells during Mtb infection are Toll-like receptors (TLRs), C-type lectins, class B scavenger receptors and the cytosolic nucleotide-binding oligomerization domain receptors (NODs) (Fig. 2) (Aqdas et al., 2021; Goyal et al., 2016; Gunther and Seyfert, 2018). In terms of direct orthologs of these in Dd (Fig. 2), only class B scavenger receptors and cytosolic proteins containing the toll/interleukin 1 receptor (TIR) domain have been identified and studied (Chen et al., 2007; Sattler et al., 2018).

TLRs, located at the plasma membrane (e.g. TLR1, TLR2, TLR4 and TLR6) or within intracellular endosomal compartments (TLR8 and TLR9), trigger signalling pathways upon ligand interaction. When the leucine-rich repeats (LRRs) of TLRs interact with their respective ligands, the cytoplasmic TIR domain triggers signalling to recruit adaptor proteins (Varshney et al., 2022). Dd lacks TLR orthologs, but it does have 45 LRR transmembrane proteins without a cytosolic TIR domain, which have been described but not further studied (Cosson and Soldati, 2008). However, there are two cytosolic proteins in Dd that contain TIR domains, TirA and TirB (Li et al., 2009). TirA expression is upregulated after *L. pneumophila* infection and is essential for the efficient phagocytosis of Gram-negative bacteria (Chen et al., 2007; Zhang et al., 2016b). However, little is known during mycobacterial infection.

In macrophages, C-type lectin receptors, such as mannose receptors or dectin-1 (also known as CLEC7A), are involved in recognizing specific carbohydrates, such as TDM, and β-glucans present in the mycobacterial cell wall, respectively. They initiate the host immune response and mannose receptors also facilitate mycobacteria internalization (Stamm et al., 2015). In Dd, three orthologs of these receptors exist, although their roles require further study (Cosson and Soldati, 2008). In mammals, three different scavenger receptors are crucial during Mtb infection: CD36, MARCO and class A scavenger receptors (SRA) (Stamm et al., 2015). Dd does not have MARCO or SRA, but three class B scavenger receptors are present. LmpA/LmpC and LmpB are functional counterparts of LIMP-2 (also known as SCARB2) and CD36 in mammals, respectively. LmpB is primarily located in lipid rafts at the plasma membrane and early phagosomes, and its absence is linked to reduced mycobacteria uptake. LmpA is predominantly present in endosomes and lysosomes, and its absence results in decreased acidification and proteolysis in phagosomes, resembling the function of LIMP-2 (Sattler et al., 2018).

CR3 is an integrin heterodimer composed of CD18 (ITGB2) and CD11B (ITGAM), with roles in chemotaxis and phagocytosis of *Mycobacterium kansasii*, *Mycobacterium smegmatis* or Mtb in both neutrophils and macrophages. Following CR3 activation, its CD18 cytoplasmic tail interacts with the F-actin cytoskeleton to facilitate phagocytosis (Smirnov et al., 2023). In Dd, ‘similar to integrin-β A’ (SibA) shares characteristics with the integrin-β chain of CR3 and is involved in adhesion and phagocytosis (Cosson and Soldati, 2008). SibB, SibC, SibD and SibE present in Dd have not yet been studied in detail. In Dd, a Venus-trap G protein-coupled receptor, Far1, is also implicated in binding bacterial PAMPs, such as folate and lipopolysaccharides, and serves as a phagocytic receptor (Xu et al., 2021).

Remarkably, amoebae have evolved a plethora of specialized and redundant receptors to engulf a variety of bacteria and interact with various surfaces. Therefore, when single receptor genes are genetically inactivated in Dd, their loss does not significantly affect cellular phenotypes due to the functional redundancy and compensatory mechanisms that exist within extended receptor families.

### Actin rearrangement

Phagocytes and Dd reorganize their actin cytoskeleton to engulf mycobacteria (Fig. 1) (Song et al., 2018). Indeed, maintaining the integrity of the actin cytoskeleton is crucial for *M. smegmatis* entry into human macrophages (Dutta et al., 2022). In Dd, the regulation of small GTPases, such as Ras and Rac, through the multidomain protein RGBARG (RCC1, RhoGEF, BAR and RasGAP-containing protein) is responsible for generating large macropinosomes that facilitate the engulfment of objects with complex shapes, such as mycobacteria (Buckley et al., 2020).

Following phagocytosis, actin facilitates the fusion of early endosomes with phagosomes. However, pathogenic mycobacteria, such as Mm, Mtb and *M. avium*, can disrupt the F-actin network of the host cell, preventing phagosome acidification and maturation, a phenomenon that is not observed with non-pathogenic mycobacteria, such as *M. smegmatis* (Guerin and de Chastellier, 2000). Similarly, in the Dd–Mm model, Mm hinders the actin nucleation-promoting activity of the WASH complex, thereby favouring phagosome maturation arrest and MCV biogenesis (Kolonko et al., 2014).

### Remodelling of membrane identity for MCV maturation

**Fig. 3.**
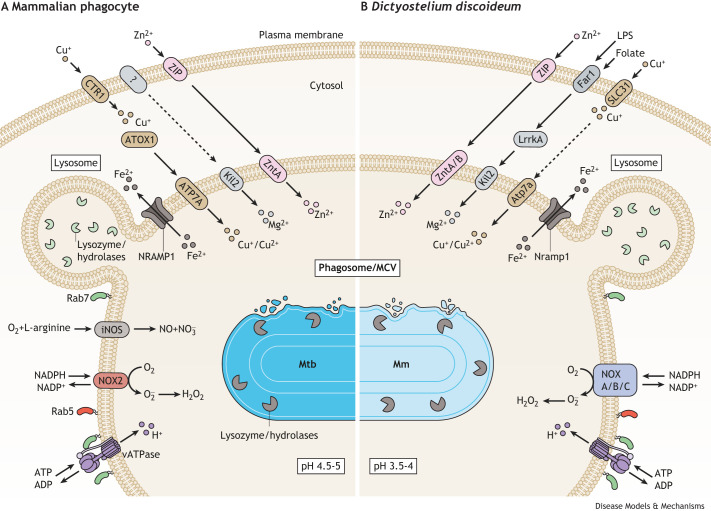
**Mammalian phagocyte and *Dictyostelium discoideum* responses to mycobacteria.** Host cell phagosomes harbour an array of mechanisms to eliminate bacteria in general, including avirulent or attenuated mycobacteria. To survive and proliferate, virulent mycobacteria must disarm this bactericidal compartment and tailor a permissive mycobacteria-containing vacuole (MCV). Phagosome mechanisms of mammalian phagocytes are shown on the left (A), and those of *Dictyostelium discoideum* (Dd) on the right (B). In both mammalian phagocytes and Dd, phagosomal vacuolar ATPase (vATPase) induces proton influx to acidify phagosomes. Concurrently, NADPH oxidase 2 (NOX2) in mammalian phagocytes and NADPH oxidase (NOX) A/B/C in Dd generates reactive oxygen species (ROS), including H_2_O_2_. Expression of inducible nitric oxide synthase (iNOS) is exclusive to mammalian phagocytes. When lysosomes fuse with phagosomes, their enzymes (e.g. lysozyme and hydrolases) selectively digest components of the mycobacteria cell wall. Mammals and Dd also both elevate the concentration of copper (Cu^+^), magnesium (Mg^2+^) and zinc (Zn^2+^) ions to poison intracellular pathogens. Copper is assimilated into the cell by copper transport protein 1 (CTR1) in mammalian phagocytes and SLC31 copper transporters in Dd. In mammals, it is transported through the cytosol by ATOX1 and incorporated into phagosomes and MCVs via ATP7A. In Dd, how Cu^+^ reaches the phagosomes remains unknown but Atp7a facilitates its translocation there. In macrophages, Kil2 facilitates Mg^2+^ accumulation in phagosomes, whereas in Dd, Mg^2+^ enters the cytosol via activation of Far1, a G protein-coupled receptor, which also recognizes bacterial lipopolysaccharides (LPS). ZIP transporters transport extracellular Zn^2+^ into macrophages and Dd, and zinc transporters (ZnTs) incorporate it into phagosomes and MCVs. Both mammalian phagocytes and Dd can also limit bacterial access to iron (Fe^2+^) through NRAMP1/Nramp1. Abbreviations: Mm, *Mycobacterium marinum*; Mtb, *Mycobacterium tuberculosis*; NADP^+^, nicotinamide adenine dinucleotide phosphate (oxidized form); NADPH, nicotinamide adenine dinucleotide phosphate (reduced form).

Remodelling of phosphatidylinositol phosphates (PIPs) in host endomembranes is evident in both macrophages and Dd infected with mycobacteria, showcasing the conserved role of PIPs during the phagocytic process (Fig. 3). Phosphatidylinositol 3-phosphate (PI3P) is a crucial regulator of phagosome maturation (Dickson and Hille, 2019; Dormann et al., 2004). After phagocytes and Dd ingest bacteria, there is an initial increase in phosphatidylinositol (3,4,5)-trisphosphate [PI(3,4,5)P3] at the engulfment site, which is rapidly converted into phosphatidylinositol (3,4)-bisphosphate [PI(3,4)P2] and PI3P, which is important for recruiting PI3P-binding proteins, such as early endosome antigen 1 (EEA1), FYVE-type zinc finger-containing PIP kinase (PIKfyve), PROPPINs and hepatocyte growth factor-regulated tyrosine kinase substrate (Hrs) (Lawe et al., 2002; Schnettger et al., 2017; Tornero-Ecija et al., 2022; Vines et al., 2023). PI3P is finally converted by PIKfyve to phosphatidylinositol (3,5)-bisphosphate [PI(3,5)P2], which drives the accumulation of Rab7 and other lysosomal machinery components on phagosomes in animal cells and Dd (Vines et al., 2023).

During infection, Mtb and Mm secrete three phosphatases: protein tyrosine phosphatase A (PtpA) and B (PtpB) and PI3P acid phosphatase M (SapM), which collectively impede phagosome maturation and promote cytosol escape from the MCV (Bach et al., 2008; Koliwer-Brandl et al., 2019; Puri et al., 2013; Saikolappan et al., 2012; Vergne et al., 2014; Wong et al., 2011). Notably, BCG also secrets SapM via the SecA2 secretion system (Xander et al., 2024).

### Rab GTPases – orchestrators of phagosome maturation

Rab GTPases play a pivotal role in orchestrating vesicle trafficking. In both Dd and mammals, PI3P on the MCV facilitates the transition from Rab5 to Rab7, which is essential for vesicular trafficking and for phagosome maturation. In Dd, Rab5A has been identified on the Mm MCV (Barisch et al., 2015) and on *Legionella*-containing vacuoles (Hoffmann et al., 2014). Also, in Dd, the pleckstrin homology (PH) domain-containing protein PripA forms a complex with TbcrA, which also promotes the conversion from Rab5A to Rab7A and contributes to phagosome maturation (Tu et al., 2022).

In macrophages infected with Mtb, Rab8A is phosphorylated by the LRR kinase 2 (LRKK2) and is recruited to early endolysosomes, leading to the recruitment of ESCRT components (Herbst et al., 2020). Although data are lacking for mycobacteria-infected Dd, LrrkA, the likely homolog of LRRK2 in Dd, influences intraphagosomal killing of *K. pneumoniae* (Bodinier et al., 2021).

Rab20, an interferon γ (IFN-γ or IFNG)-inducible GTPase, associates rapidly with phagosomes in mycobacteria-infected macrophages to maintain MCV integrity and reduces the cytosolic translocation of mycobacteria (Egami and Araki, 2012; Schnettger et al., 2017; Seto et al., 2011). Notably, Rab20, Rab5C and Rab11B are upregulated in sputum samples from patients with active TB. However, Mtb can trigger Rab20 dissociation, disrupting phagosome maturation and ensuring its survival (Schnettger et al., 2017). In Dd, no Rab20 ortholog has been identified.

PE_PGRS proteins represent some of many virulence factors produced by mycobacteria. Mtb deploys virulence factors that regulate membrane trafficking (Chai et al., 2020), such as PE_PGRS20 and PE_PGRS47, which directly bind to Rab1A, blocking autophagy initiation (Strong et al., 2021). Moreover, the nucleoside diphosphate kinase (NdK) associated with the inactivation of both Rab5 and Rab7 further contributes to Mtb evasion mechanisms (Sun et al., 2010). Mycobacteria have developed numerous other virulence factors to counteract the host defence mechanisms, as elaborated below.

## Host defence mechanisms and mycobacterial counterattacks

The phagosome, which initially houses engulfed mycobacteria before evolving into an MCV, acts as a time bomb that is armed with various mechanisms to eliminate the pathogen (Fig. 3). However, mycobacteria have evolved various strategies to counteract these host defence mechanisms, as we discuss in this section.

### Chemical warfare – pH acidification, ROS and RNS

The maintenance of an acidic pH in phagosomes is an important defence mechanism against infection. Macrophage phagosomes typically have a pH of 4.5-5, whereas Dd phagosomes have a pH lower than 3.5 (Marchetti et al., 2009). A recent study suggests that the pH of phagosomes can also fluctuate in response to the nature of the cargo and external stimuli (Foote et al., 2019).

The vacuolar ATPase (vATPase) plays a crucial role in the acidification of mammalian and Dd phagosomes by pumping protons in their lumen (Soldati and Neyrolles, 2012). Moreover, neutrophils and macrophages possess the H^+^ channel Hv1 (or HVCN1) for further phagosome acidification (El Chemaly and Demaurex, 2012), but it has not been yet described in Dd. Proton accumulation is counteracted by chloride influx and cation efflux of the host (Soldati and Neyrolles, 2012). Remarkably, Mtb sheds the antacid 1-tuberculosynyladenosine (1-TbAd) to arrest lysosomal acidification (Bedard et al., 2023; Buter et al., 2019).

NADPH oxidase 2 (NOX2, also known as gp91^phox^ or CYBB) generates the superoxide anion (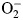), which is released into the phagosomal lumen and is transformed into other reactive oxygen species (ROS). In mammals, the NADPH oxidase (NOX) complex consists of the catalytic transmembrane protein NOX2 and the regulatory subunit p22^phox^ (also known as CYBA). In Dd, there are three functional homologs of NOX catalytic subunits: NoxA and NoxB, which are NOX2 homologs, and NoxC, which is a NOX5 homolog. Dd also encodes a regulatory subunit, CybA (p22^phox^ in mammals) and the cytosolic activating factor NcfA (p67^phox^ or NCF2 in mammals) (Zhang et al., 2016b). Although the major source of ROS is NOX2, it can also be produced by the respiratory electron transport chain of mitochondria (Dan Dunn et al., 2015) and by peroxisomes (Huang et al., 2023). ROS contribute to better control of Mtb in macrophages (Chandra et al., 2020). Furthermore, peroxisomal ROS limits the cytosolic growth of Mtb in macrophages derived from Mtb-infected human IPSDMs (Pellegrino et al., 2023). In Dd, NoxA is crucial for eliminating *K. pneumoniae* (Crespo-Yanez et al., 2023) and for lysing *P. aeruginosa* (Ayadi et al., 2024). However, it is unknown whether ROS play a role in Dd cell-autonomous defences during mycobacterial infection, necessitating further investigation.

Increased ROS levels impact both pathogens and host cells, leading to the generation of ROS-detoxifying enzymes by hosts, including superoxide dismutases (SODs), catalases and peroxiredoxins. Mtb has also evolved mechanisms to counteract ROS production by producing SODs (Edwards et al., 2001), by inhibiting ROS production (Pellegrino et al., 2023), by reducing NOX2 expression (Lv et al., 2017) or by modulating host NOX2 activity (Koster et al., 2017; Mo et al., 2022). In response to oxidative stress, mycobacterial SodA, mycolic acids, KatG and SodC may help mitigate the toxicity of extracellular ROS (Tyagi et al., 2015). Additional protective strategies include the use of mycobacterial cytosolic reducing buffers, such as mycothiol and thioredoxins, to maintain the redox environment within the bacteria (Pacl et al., 2018). Moreover, Mtb possesses a type I NADH dehydrogenase that antagonizes phagosomal NOX2 activity (Miller et al., 2010), and Mtb also secretes CpsA, which interferes with NOX activity, thereby reducing ROS concentration in phagosomes and impeding its clearance (Koster et al., 2017).

Neutrophils and macrophages also release extracellular traps in response to pathogens, including in response to Mtb, which affects its course of infection (Garcia-Bengoa et al., 2023). In multicellular Dd slugs infected with *K. pneumoniae* or exposed to lipopolysaccharide, sentinel cells can produce extracellular traps, which is dependent on ROS produced by NoxA, NoxB and NoxC (Zhang et al., 2016b). However, further research is required to determine the role of extracellular traps during mycobacterial infection in Dd.

Reactive nitrogen species (RNS), such as nitric oxide (NO), and inducible NO synthase (iNOS, also known as NOS2, Box 1) are crucial components of the innate immune responses against mycobacterial infections, as reviewed by Yang et al. (2009) and Jamaati et al. (2017). Mtb-infected mouse and human macrophages induce iNOS production to significantly different levels *in vitro*. Indeed, human macrophages generate lower levels of NO than mouse macrophages, which complicates our understanding of iNOS in Mtb control (Jung et al., 2013). Furthermore, NO can direct macrophages to form multinucleated giant cells, which create a permissive environment for mycobacterial persistence (Gharun et al., 2017). It is worth noting that Dd has no recognizable NO synthase, precluding the study of NO during mycobacterial infections.

### Lysosomal enzymes

The process of phagosome maturation into a degradative and bactericidal milieu is conserved between mammalian cells and Dd (Boulais et al., 2010). The lysosome fuses with the phagosome following Rab7 interaction with members of the homotypic fusion and protein sorting (HOPS) complex, such as Vps18 (Jani et al., 2023 preprint), which then functions as a tethering complex involved in vesicle trafficking. Following this, lysosomal enzymes are delivered to phagosomes to target specific components of the cell wall and membranes of the pathogen (Trivedi et al., 2020).

Mtb employs multiple evasion strategies at this stage. It upregulates the lipoprotein LprI to neutralize lysozyme (Box 1) activity in peritoneal and monocyte-derived macrophages (Sethi et al., 2016), and it expresses PE_PGRS proteins (Iantomasi et al., 2012; Saini et al., 2016) and EsxA (also known as ESAT-6) (Xu et al., 2007; Tan et al., 2006) to inhibit phagosome maturation. Additionally, Mtb exploits lysosome-poor monocyte-derived cells for persistence *in vivo* (Zheng et al., 2024).

In the Dd model, the membrane-permeabilizing proteins AlyL and BpiC, which target peptidoglycans and lipopolysaccharides, respectively, are effective against *K. pneumoniae* (Crespo-Yanez et al., 2023), but it is not yet known if these are effective against mycobacteria. Furthermore, in Dd, Kil1 (Box 1) and Kil2 (Box 1) play vital roles in bacterial digestion within phagosomes. Kil1 delivers proteases (Bodinier et al., 2020), whereas Kil2, activated by folate, enhances magnesium ion transfer to the phagosomal lumen, improving lysosomal enzyme efficiency. Both Kil1 and Kil2 contribute significantly to *K. pneumoniae* digestion (Crespo-Yanez et al., 2023), although their roles in mycobacterial infection remains unexplored.

### Metal transporters

The regulation of essential divalent metals, such as zinc, copper, iron and magnesium, as enzyme cofactors, is crucial for both hosts and pathogens. Host cells employ strategies to either poison intracellular pathogens or to deprive them of essential micronutrients.

Zinc is an abundant micronutrient that is crucial for regulating gene expression, cell processes, immune responses and/or antioxidant defences, among other roles in the host. Indeed, about 10% of the human proteome present with zinc-binding motifs (reviewed by Maret, 2013). In eukaryotes, ZIP family transporters facilitate extracellular zinc entry into the cell cytosol, whereas the zinc transporter (ZnT) proteins export zinc outside the cell or to the lumen of endocytic and secretory organelles. In Dd, seven ZIP transporters (ZplA-G) and four ZnT transporters (ZntA-D) have been identified (reviewed by Dunn et al., 2018). Upon Mm infection of Dd cells, the ZntA and ZntB efflux pumps are recruited to the MCV, increasing the zinc concentration in MCVs and restricting mycobacteria growth (Barisch et al., 2018; Hanna et al., 2021), as also observed in macrophages infected with Mtb (Botella et al., 2011; Neyrolles et al., 2013). Mycobacteria counteract zinc poisoning with metal efflux pumps, including CtpC in Mtb and Mm, and CtpG in BCG (Boudehen et al., 2022; Chen et al., 2022; Hanna et al., 2021).

Copper, a redox-active metal, undergoes cycles between the Cu^+^ and Cu^2+^ ion states in the MCV under physiological conditions. Mammalian copper transport protein 1 (CTR1) pumps copper into the cytosol, where it binds ATOX1, delivering it to ATP7A present on the MCV membrane (reviewed by Neyrolles et al., 2015). Dd expresses six copper transporters, including three SLC31 copper transporters and three P-type Cu-ATPases, one sharing homology with human ATP7A (Buracco et al., 2018). In mammalian phagocytes and Dd, the recruitment of ATP7A to phagosomes might enhance copper pumping into the phagosomal lumen, whereas p80, a predicted copper transporter homolog of CTR1, might play a role in copper influx to the cytosol (Buracco et al., 2018; Neyrolles et al., 2015). Mycobacteria deploy defences against copper stress. They express Cu^+^-binding metallothionein (MymT), copper transport protein B (MctB) and copper (I) transporting P_1B_-type ATPases (CtpV and CtpB) (reviewed by Botella et al., 2012; Darwin, 2015; Leon-Torres et al., 2020). Experimental use of copper chelators on Mtb-infected macrophages reduced bacterial load, indicating a potential role for copper in Mtb intracellular growth (Libardo et al., 2018; Shah et al., 2016; Speer et al., 2013). However, copper fluctuations during Dd growth did not affect *Legionella* infection (Buracco et al., 2018), and similar research is needed for mycobacteria.

Nutritional immunity refers to host strategies that impede pathogen growth by limiting metal availability. In Mtb-infected macrophages, the host protein NRAMP1 diminishes the availability of iron by redirecting its storage from the phagolysosome to the cytosol (reviewed by Murdoch and Skaar, 2022). NRAMP1 is expressed both by macrophages and Dd and appears to restrict the growth of mycobacteria (Medapati et al., 2017; Peracino et al., 2006). Mammals also produce NRAMP2 (also known as SLC11A2), but Dd Nramp2 is more akin to protist and fungal Nramp proteins (Peracino et al., 2013). Dd Nramp2 restricts *Francisella* growth (Brenz et al., 2017), whereas data for mycobacteria are still lacking. The iron response of mycobacteria involves siderophores (Box 1), which facilitate their growth in both Dd and mouse macrophages (Knobloch et al., 2020). In the case of Mtb and Mm, distinct siderophores, mycobactin and carboxymycobactin, have been identified. For a comprehensive review of the relevance of iron to TB pathogenesis, see Rodriguez et al. (2022).

### Host metabolism

Target of rapamycin complex 1 (TORC1) (Box 1) inhibits autophagy, promoting animal and Dd cell growth by boosting ribosome biogenesis and protein translation in nutrient-rich conditions. However, under conditions of low nutrients, TORC1 is inhibited, and autophagy provides the metabolites and energy required to sustain essential functions in both mammalian cells and Dd (Cardenal-Muñoz et al., 2017; Linares et al., 2013).

Upon Mm infection, the mammalian target of rapamycin (mTOR) kinase is inhibited, resulting in a host-protective effect by enhancing autophagy and glycolysis in Dd and zebrafish larvae that lack adaptive immunity, relying solely on innate responses (Cardenal-Munoz et al., 2018; Pagán et al., 2022). Similar responses have been observed in Mm- and Mtb-infected THP-1 macrophages (Pagán et al., 2022). Moreover, in mammalian cells, the interaction of stress granule (Box 1) proteins, such as NUFIP2 or G3BP1, with GABA receptor-associated proteins (GABARAPs) ensures the inactivation of mTORC1 via the Ragulator–Rag system (Jia et al., 2022)*.* However, mTOR deficiency can also lead to a significant innate susceptibility to mycobacteria, leading to the death of infected macrophages through (or due to) elevating mitochondrial energy metabolism driven by glycolysis in response to infection (Pagán et al., 2022).

Experiments in Dd have shown that Mm blocks autophagic flux, resulting in the accumulation of membranes and cytoplasmic material in the MCV, potentially supporting bacterial survival within this niche (Cardenal-Muñoz et al., 2017).

## Host resilience

During infection, the slightly acidified MCV milieu activates the membranolytic factor EsxA of Mtb and Mm (Bao et al., 2021). This, coupled with other virulence factors, such as PDIMs, contribute to MCV damage (Augenstreich et al., 2017; Bussi et al., 2023; also reviewed by Augenstreich and Briken, 2020; Chandra et al., 2022). As observed in Dd, the membranolytic activity of these mycobacterial virulence factors benefits from membrane microdomains that contain sterols and vacuolins, which are homologs of mammalian lipid raft-associated flotillins (Box 1) (Bosmani et al., 2021 preprint). Similarly, Mtb can also cause lysosomal damage, triggering protease leakage into the cytosol and leading to mitochondrial disruption (Bhattacharyya et al., 2023; Bussi et al., 2022; Radulovic et al., 2022). Such damage interferes with cellular functions and activates immune responses. As a result, cells employ various mechanisms to recognize and limit damage to their membranes caused by intracellular pathogens. Although the characteristics that determine which repair pathway responds to membrane damage are still poorly understood, the extent of the damage appears to be crucial for activating repair mechanisms. Minor damage to membranes is repaired by the ESCRT system (Radulovic et al., 2018) or through membrane contact sites (Radulovic et al., 2022). More extensive damage to host cell membranes induces the activation of autophagy (Schnettger et al., 2017).

In this section, we review and discuss the initial events that follow mycobacterial-induced host membrane damage, highlighting the mechanisms employed by host cells to repair the damage and how these repair machineries are coordinated (Fig. 1).

### Sensing mycobacteria-triggered damage

Host galectin-3 (LGALS3), galectin-8 (LGALS8) and galectin-9 play vital roles in recognizing host glycolipids and glycoproteins on the luminal leaflet of the MCV that become exposed to the cytosol after Mtb-induced membrane damage (Bell et al., 2021).

ESX1-dependent damage triggered by Mtb induces the recruitment of galectin-3 in iPSDMs and THP-1-infected cells (Fig. 1) (Augenstreich et al., 2017; Beckwith et al., 2020). Additionally, in bone marrow-derived macrophages, galectin-9 binds to the cytosolically exposed arabinogalactan of the Mtb cell wall (Morrison et al., 2023; Wu et al., 2021). In macrophages, galectin-8 might play a direct role in the repair and clearance of Mtb-induced MCV damage (Jani et al., 2023 preprint), possibly due to its role in recruiting the autophagy machinery (Bell et al., 2021). Overall, although galectins are involved in damage sensing, they do not seem to limit mycobacteria growth (Morrison et al., 2023). Dd lacks galectin orthologs but possesses functionally homologous discoidins (DscA, DscC, DscD and DscE), emphasizing the evolutionary adaptations in response to mycobacterial infections in different hosts (Aragao et al., 2008; Mathieu et al., 2010). Cytosolic discoidins recognize glycosylated Mm lipids or proteins exposed to the cytosol after MCV rupture (Fig. 1). Specific ligands for discoidins in the Mm cell wall and the implications of their recognition are still under investigation (López-Jimenez, 2017).

### Ubiquitin as a **‘**repair-me’ and ‘eat-me’ signal

Ubiquitination (Box 1) is a process conserved between animal cells (Madiraju et al., 2022) and Dd (Pergolizzi et al., 2019; Raykov et al., 2023; Xiong et al., 2023) and is important in Mtb-infected human macrophages and Mm-infected Dd. Smurf1 transfers lysine (K) 48-linked ubiquitin chains that serve as a signal for degradation by the proteasome, and parkin proteins transfer K63-linked ubiquitin chains recognized by autophagy adapters (Franco et al., 2017; Manzanillo et al., 2013). Additionally, essential E3 ubiquitin ligases such as tumour necrosis factor α (TNF-α) receptor-associated factors (TRAFs) and tripartite motif-containing proteins (TRIMs) also contribute to infection outcomes.

In humans, seven TRAFs have been characterized (TRAF1-7). TRAF6, a RING-type E3 ligase, influences various host immune defence functions, such as the transcription of TNF-α (Kim et al., 2022b) and transforming growth factor β (TGF-β, encoded by *TGFB*) (Landstrom, 2010), the induction of autophagy (Kim et al., 2022a) or the maturation of autophagosomes under oxidative stress (Wang et al., 2022).

In Dd, over 40 TRAF-like proteins have been predicted, with 16 of them presenting RING, zinc finger and TRAF domains akin to those of mammalian TRAF2, TRAF3, TRAF5 and TRAF6 (Dunn et al., 2018). In particular, the TRAF E3 ubiquitin ligase TrafE (a TRAF6 ortholog) plays a pivotal role during Mm infection in Dd. Specifically, TrafE is proposed to act as a coordinator between ESCRT and autophagy pathways through TrafE-mediated K63 ubiquitination of yet unknown target(s) (Raykov et al., 2023).

TRIM proteins are a conserved ubiquitin ligase family that have diverse roles in immune responses or autophagy. The expression of 20 TRIM genes in patients with active TB was reported to be decreased compared to that in patients with latent TB or healthy donors, linking these genes to the pathogenesis of TB and highlighting their potential utility as TB biomarkers (Chen et al., 2018). Mammalian TRIM proteins have been linked to Mtb (TRIM16, TRIM22, TRIM27 and 32), are shown to induce autophagy (TRIM16, TRIM22 and TRIM32) (Chauhan et al., 2016; Lou et al., 2018; Romagnoli et al., 2023) and are counterintuitively associated with Mtb growth (TRIM14, TRIM25, TRIM36 and TRIM56) (Hoffpauir et al., 2020). Dd possesses a single TRIM protein (Dunn et al., 2018) with homology to human TRIM37, associated with autophagy and viral restriction (Gu et al., 2023). However, the specific role of Dd TRIM in mycobacterial infection remains understudied (Raykov, 2021).

### Influx of extracellular Ca^2+^

In eukaryotes, efficient membrane repair often relies on the influx of Ca^2+^ (Gronski et al., 2009; Yuan et al., 2001), triggering the accumulation of calcium-binding proteins at the wound site to support cytoskeletal reorganization and the assembly of signalling molecules, as reviewed by Cooper and McNeil (2015).

During Mtb phagocytosis, intracellular Ca^2+^ levels rise in response to opsonized or heat-killed Mtb, whereas in response to live Mtb, intracellular Ca^2+^ levels decrease in macrophages, affecting proteins such as calmodulin and phosphorylated Ca^2+^/calmodulin-dependent protein kinase II (CaMKII) (Jayachandran et al., 2007). In Mtb-infected macrophages, intracellular Ca^2+^ signalling is crucial for membrane trafficking, which involves LRRK2, Rab8 and ESCRT machinery recruitment at damaged endolysosomes (Herbst et al., 2020).

Upon sterile damage in Dd, accumulation of actin filaments at the wound site relies on Ca^2+^ influx, which is crucial for repair (Talukder et al., 2020). However, no study is available relating calcium and mycobacteria infection in the amoeba model.

Remarkably, mycobacterial virulence factors, such as mannose-capped LAM (ManLAM) (Rojas et al., 2000), PE6 (Medha et al., 2023), the secreted protein PE_PGRS1 that contains seven Ca^2+^ binding domains (Yu et al., 2023), and the calcium P-type ATPase CtpF (Maya-Hoyos et al., 2022), thwart host intracellular Ca^2+^ increase to ensure bacteria survival.

### Membrane-damage repair mechanisms

Host cells respond to membrane damage through coordinated repair strategies that are influenced by the extent and characteristics of the damage. In this subsection, we discuss primary repair mechanisms in mammalian macrophages and Dd and shed light on recent insights into the membrane damage that is induced by mycobacteria.

Small membrane damage in the MCV of Mtb-infected macrophages (Philips et al., 2008) and of Mm-infected Dd (Lopez-Jimenez et al., 2018) is primarily repaired by ESCRT to ensure bacterial containment, highlighting the conservation of this repair pathway (Fig. 1). In Mm-infected Dd, ESCRT proteins localize to the MCV, forming distinct patches or rings. In response, Mtb employs a countermeasure by secreting EsxG and EsxH factors through the ESX-3 type VII secretion system to hinder the recruitment of the ESCRT-III machinery to sites of MCV damage (Mittal et al., 2018).

ESCRT is not limited to endolysosomal repair; it also contributes to plasma membrane repair. In Mtb-infected THP-1 cells, damage-induced calcium influx triggers the recruitment of ALG2 to the plasma membrane (Beckwith et al., 2020). In TR146 mammalian cells infected with *Candida albicans*, ALG2 then interacts with ALG2-interacting protein X (ALIX, also known as PDCD6IP) and with CHMP proteins of the ESCRT-III complex, facilitating plasma membrane repair (Westman et al., 2022). This phenomenon is similar in Dd, where PefA, one of the two penta-EF hand homologs of mammalian ALG2, and ALIX are recruited to the site of plasma membrane damage in a calcium-dependent manner (Talukder et al., 2020).

Autophagy is active in both mammals and Dd, which possesses orthologs of most autophagy-related mammalian proteins, as reviewed by Calvo-Garrido et al. (2010), Cosson and Soldati (2008) and Mesquita et al. (2017). In Mm-infected Dd, an increased number of Atg8- and Atg18-positive structures, along with the recruitment of autophagy markers, such as ubiquitin and p62, are observed soon after infection (Cardenal-Muñoz et al., 2017).

Recent findings propose that mammalian ATG8 proteins bind to ESCRT components to maintain the integrity of autophagosomes (Javed et al., 2023). Autophagy-mediated membrane repair is exemplified by the presence of Vps32 (also known as CHMP4), Atg8 and the autophagy adaptor p62 (SQSTM1) at pathogen-containing vacuoles in mammalian cells infected with *Salmonella* (Kreibich et al., 2015) or *C. albicans* (Lapaquette et al., 2022), or in Dd infected with Mm (Lopez-Jimenez et al., 2018; Raykov et al., 2023) or *A. fumigatus* (Ferling et al., 2020).

In the Dd–Mm model, the ESX-1 secretion system of Mm induces a robust repair response involving both the ESCRT-III and autophagy pathways (Lopez-Jimenez et al., 2018; Raykov et al., 2023). The coordination of both pathways by TrafE has been demonstrated in the Dd–Mm model (Raykov et al., 2023), and it is thus plausible that a similar scenario occurs in Mtb-infected mammalian cells.

Recent research in mammalian cells has revealed additional cellular mechanisms that mend damaged membranes, notably involving membrane–contact sites and the formation of stress granules (Bussi et al., 2023). Following lysosomal damage, phosphatidylinositol 4-kinase type 2-α (PI4K2A) orchestrates the synthesis of phosphatidylinositol 4-phosphate (PI4P), resulting in its excessive accumulation on the injured vacuole. This buildup triggers the recruitment of oxysterol-binding protein (OSBP), an endoplasmic reticulum (ER)–Golgi protein, and OSBP-related proteins (ORPs) 9-11 (also known as OSBPL9-11), giving rise to extensive ER–lysosomal membrane contact sites. OSBP plays a crucial role in transferring cholesterol and phosphatidylserine from the ER to damaged lysosomes, reciprocated by PI4P, thereby regulating PI4P levels. Likewise, ORPs, with their lipid binding and transport capabilities, facilitate PI4P-driven phosphatidylserine transfer from the ER to damaged lysosomes, mirroring the functions of ORP1L (an isoform of ORP1, also known as OSBPL1A), ORP5 (OSBPL5) and ORP8 (OSBPL8) (Chung et al., 2015; Radulovic et al., 2022; Tan and Finkel, 2022). Notably, OSBP is recruited to Mtb and Mm-containing vacuoles in an ESX-1-dependent manner. In Dd, OSBP8 is also recruited early during infection and is present on ER tubules forming contact sites with the MCV. OSBP8 depletion negatively impacts Dd cell viability and enhances Mm growth (Anand et al., 2023).

In human macrophages infected with Mtb, stress granules and other condensates rapidly nucleate nearby damaged MCVs or endolysosomes, acting as a plug to stabilize ruptured membranes. The complete engulfment of the droplet inside the compartment aids in its effective repair, either spontaneously or facilitated by the ESCRT machinery (Bussi et al., 2023). Notably, mammalian ATG8 proteins can interact with stress granule proteins, influencing stress granules and mTOR responses to Mtb damage (Jia et al., 2022), indicative of a coordinated cellular response. Although Dd induces protein aggregation under stress, the specific role of these protein condensates as potential patches upon mycobacteria-induced membrane damage remains unknown.

### Mycobacteria restriction

Extensive research has been conducted on xenophagy, a specific form of autophagy that targets intracellular pathogens, to understand its role in controlling Mtb infection and in limiting its growth in macrophages (Gutierrez et al., 2004). This phenomenon has been observed in various models, including in Dd–Mm, BMDM–Mm and iPSDM–Mtb, demonstrating its conservation between amoebae and mammals (Bernard et al., 2021; Collins et al., 2009; Lopez-Jimenez et al., 2018; Simeone et al., 2012). Xenophagy activation involves galectin recruitment and ubiquitination of the bacteria at damaged MCVs (Fig. 1). It can also be initiated by ubiquitinated bacteria in the cytosol or be enhanced by cGAS–STING-dependent signalling, which recognize foreign DNA in the cytosol (Bernard et al., 2021; Watson et al., 2015, 2012).

The deletion of autophagy-related genes in mouse macrophages *in vivo* is linked to increased levels of cytosolic Mtb and Mm, leading to rapid necrotic cell death (Feng et al., 2024; Golovkine et al., 2023). Specifically, knocking out autophagy-related genes, such as ATG7, ATG14 and ATG16, increased Mtb growth and subsequent macrophage cell death. Although xenophagy triggers a type I interferon immune response in mammals to restrict mycobacteria growth, some authors propose that mycobacterial control is mainly achieved by promoting phagosome maturation and by activating the autophagy machinery (Schnettger et al., 2017). Accordingly, Atg8a (an LC3 family protein commonly used as autophagy reporter) is observed at MCVs in the Dd–Mm model (Cardenal-Muñoz et al., 2017; Lopez-Jimenez et al., 2018; Raykov et al., 2023) and in mammalian macrophages infected with Mtb (Bernard et al., 2021). Additionally, the autophagy regulator DRAM1 forms puncta near mycobacteria, resulting in the colocalization of DRAM1, LC3 and Mm in zebrafish and macrophages (Banducci-Karp et al., 2023). However, deciphering the specific contribution of autophagy-related genes to mycobacteria control poses challenges, particularly given the contrasting findings related to the knockout of the ATG5-coding gene (Castillo et al., 2012; Golovkine et al., 2023; Kimmey et al., 2015; Kinsella et al., 2023; Watson et al., 2012). Overall, the consensus in the field is that autophagy is the primary pathway that restricts cytosolic mycobacteria, with ATG5 exhibiting additional and unique functions beyond autophagy (Deretic and Wang, 2023).

In the context of TB, guanylate-binding proteins (GBPs, Box 1) are associated with susceptibility to bacterial infection and host response (Esterhuyse et al., 2015; Kim et al., 2012), as they bind to MCVs and limit the growth of intracellular pathogens, such as BCG (Marinho et al., 2020). However, their ability to restrict the growth of Mtb does not extend to virulent Mtb expressing the ESX-1 secretion system in infected mice (Olive et al., 2023). Conversely, Mtb secretes virulence factors, such as PE31, that induce GBP1 production in macrophages and reduce macrophage apoptosis (Ali et al., 2020). The role of the single Dd homolog of human GBPs remains understudied (Raykov, 2021).

Despite host efforts to control and eliminate mycobacteria, whether contained within the MCV or in the cytosol, virulent mycobacteria can also win, proliferate and disseminate. This last phase of the productive infection cycle will be presented in the following section.

## Mycobacteria triumph and dissemination

The intracellular localization of mycobacteria cycles between the MCV and the host cytosol in a dynamic manner, reflecting the competing processes of mycobacteria-triggered damage and host repair machineries in Dd (Cardenal-Muñoz et al., 2017; Lopez-Jimenez et al., 2018; Raykov et al., 2023) as well as in mouse macrophages (Schnettger et al., 2017) and iPSDMs (Bernard et al., 2021). Host cells employ diverse mechanisms to constrain and eradicate mycobacteria. For instance, Dd initially expels intracellular bacteria by exocytosis, ejection or by host lysis; infected cells are then excluded from multicellular aggregates by a collective effort, thereby ensuring germ-free spores (López-Jiménez et al., 2019 preprint).

Despite these host defence responses, mycobacteria can persist and propagate within cells, leading to their dissemination to other cells or organisms (Fig. 1). The rupture of the phagolysosome or the cytosolic sensing of bacterial DNA activates various cell death processes that are crucial for Mtb spread (Ruan et al., 2024). In some cases, cell death can even be induced by extracellular contact with mycobacterial aggregates (Toniolo et al., 2023). These host cell death processes include non-lytic and lytic processes, such as apoptosis in mouse macrophages and hMDMs (Aguilo et al., 2013; Augenstreich et al., 2017), necrosis in BMDMs (Lee et al., 2006), necroptosis (Box 1) in mice (Pajuelo et al., 2018; Zhao et al., 2017), pyroptosis (Box 1) in THP-1 cells and peripheral blood mononuclear cells (Beckwith et al., 2020; Golovkine et al., 2023), as well as other mechanisms mediated by interferons in BMDMs (Zhang et al., 2021) and by TNF signalling in mammalian cells or zebrafish (Roca et al., 2019).

In Dd, an additional mechanism of mycobacterial dissemination involves Mm egress from the cell via a regulated process that balances host cell integrity with infection spread (Hagedorn et al., 2009). Ejection, leading to host plasma membrane damage, is controlled by an autophagosome structure in Dd, whereas Mm uses a barrel-shaped F-actin structure (ejectosome) for egress (Fig. 1). ESCRT and autophagic proteins at the distal pole shield the host cell from lysis during Mm egress (Gerstenmaier et al., 2015), with Vps4 completing membrane sealing upon bacterial exit. Despite the unknown localization mechanism of Vps4 (Gerstenmaier et al., 2024), the dependence of its recruitment on TrafE at MCV damage sites in Dd (Raykov et al., 2023) suggests some level of coordination with the response at the ejectosome site.

## Conclusion

The natural ability of Dd as a bacterial predator and its reliance on only cell-autonomous defences makes it crucial for exploring innate immunity against various microorganisms. Specifically, the synergy between Dd and Mm as a model for investigating host–pathogen interactions presents a unique opportunity. This is primarily due to the similarities in virulence factors between Mm and Mtb, as well as their infection processes in Dd and macrophages, respectively. Although Dd offers experimental advantages due to its conserved innate immune pathways, it is also essential to consider its evolutionary distance from macrophages.

To trigger initial phagocytosis of (myco)bacteria, Dd exhibits a myriad of receptors, although their genetic study is complicated by functional redundancy. Nonetheless, cytosolic proteins such as TirA are recognized for their significance in sensing pathogens other than mycobacteria. Additionally, although lectin receptors and integrin-like receptors are present in Dd, further investigation is necessary to delineate their roles during (myco)bacterial infections. After phagocytosis, mycobacteria transiently reside within phagosomes, which usually mature into the MCV, the ultimate bactericidal machine to eradicate and infection. Dd has been instrumental in elucidating the specific role of PI(3,5)P2 in Rab7 accumulation and lysosomal biogenesis. However, certain important mammalian Rabs, such as Rab20, remain unidentified in Dd.

Both mammalian phagocytes and Dd employ mechanisms such as vATPase accumulation and ROS production against pathogens. However, research gaps persist in Dd, including the identification of additional acidification channels (Hv1 H^+^ channel) and the specific role of ROS during mycobacterial infection. Although neutrophils, macrophages and Dd release extracellular traps, their involvement in response to pathogens is only understood in mammalian cells.

Phagosomal maturation involves the delivery of lysosomal enzymes into the phagosome in both macrophages and Dd. Although several lysosomal enzymes (AlyL, BPiC, Kil1 and Kil2) have been implicated in Dd infected with *K. pneumoniae*, their specific roles in the context of mycobacterial infection require further investigation. Other antibacterial mechanisms employed by Dd and macrophages consist of regulating the presence of essential metals to poison pathogens or limit their availability, with zinc and copper being particularly noteworthy in Mtb-infected macrophages. However, copper fluctuations were not considered important in *Legionella*-infected Dd, and further research is required on mycobacterial infection.

Both Mtb and Mm convert the phagosome into a more friendly MCV, in part by causing damage to the membrane surrounding the mycobacteria. Galectins are implicated in damage sensing triggered by mycobacteria in mammals, yet specific ligands of discoidins (galectin homologs) in Dd remain under intense research. Ubiquitination is also crucial during infection, with the E3 ubiquitin ligase TrafE demonstrated to be essential in coordinating repair mechanisms in Dd. Despite some similarities, this precise pathway has not yet been described in mammals. However, and reciprocally, other ubiquitin ligases such as TRIMs, implicated in mycobacterial infections in mammals, deserve further investigation in the Dd model.

Repair mechanisms, including ESCRT, autophagy, membrane contact sites and stress granules, are conserved in eukaryotes. Although ESCRT and autophagy are described in mammals and Dd in response to mycobacterial damage, the precise roles of membrane contact sites and stress granules in the Dd–Mm model remain to be elucidated.

Overall, the Dd–Mm model facilitates the elucidation of critical mechanisms in a safe and 3R (Replacement, Reduction and Refinement)-compliant manner, aiming to advance the development of effective therapies against TB infection.
